# Influence of oncogenic drivers and signaling pathways on immune checkpoint inhibitor efficacy in NSCLC

**DOI:** 10.1016/j.gendis.2026.102230

**Published:** 2026-05-12

**Authors:** Chunyan Liu, Shan Zhu, Jing Wu, Yuan Qiao, Ning Yang, Jingtao Chen

**Affiliations:** a Laboratory for Tumor Immunology, The First Hospital of Jilin University, Changchun, Jilin 130061, China; b Department of Gynecology, The First Hospital of Jilin University, Changchun, Jilin 130021, China; c Cancer Center, The First Hospital of Jilin University, Changchun, Jilin 130021, China

**Keywords:** Immunotherapy, Non-small cell lung cancer, Signaling pathway inhibitor or agonist, Tumor microenvironment, Tyrosine kinase inhibitor

## Abstract

Non-small cell lung cancer (NSCLC) is characterized by intricate genetic modifications and mechanisms that allow it to evade immune detection, which complicates the pursuit of lasting therapeutic responses. The effectiveness of treatments is greatly affected by the interaction between tumor genetic profiles and the surrounding immune microenvironment, highlighting the need for carefully designed therapeutic combinations. This review provides a comprehensive analysis of oncogenic drivers and signaling pathways (such as the *KRAS*, *BRAF*, and CD73/adenosine pathway) that interact with immune checkpoint inhibitors (ICIs), offering practical insights for personalized treatment approaches. The key findings focus on the use of biomarkers to guide the selection of immunotherapy or ICI-based combination therapies (*e.g.*, ICIs plus tyrosine kinase inhibitors or signaling pathway inhibitors). We emphasize the importance of integrating tumor genomics with immune profiling to improve patient survival outcomes. This review aims to optimize treatment strategies by synthesizing insights from recent clinical trials and preclinical research, ultimately improving therapeutic efficacy in NSCLC.

## Introduction

Lung cancer remains the leading cause of cancer-related mortality, second only to breast cancer in terms of new cases, with an estimated 1.8 million deaths (18%) and approximately 2.2 million new diagnoses (11.4%) in 2020.^1^ Non-small cell lung cancer (NSCLC) accounts for approximately 85% of lung cancer incidence, encompassing several subtypes, such as adenocarcinoma, squamous cell carcinoma, and large cell carcinoma.^2^

Before the advent of gene-targeted therapy and immunotherapy, the main treatment methods for NSCLC were surgery, chemotherapy, and radiotherapy. In 2004, gefitinib, an epidermal growth factor receptor tyrosine kinase inhibitor (EGFR-TKI), was introduced as the first gene-targeted drug for NSCLC patients harboring epidermal growth factor receptor (*EGFR*) mutations.^3^^,^^4^ Subsequently, a diverse array of tyrosine kinase inhibitors (TKIs) targeting oncogenic drivers (such as ALK-TKIs, BRAF-TKIs, and KRAS G12C-TKIs) was developed. However, the emergence of drug resistance frequently diminishes long-term patient outcomes. A pivotal 2016 randomized controlled trial demonstrated that pembrolizumab, an anti-programmed death-1 (PD-1) monoclonal antibody, significantly prolonged survival and reduced treatment-related toxicity compared to docetaxel in previously treated patients with advanced NSCLC.^5^ Together, gene-targeted therapy and immunotherapy have become cornerstone treatment strategies for NSCLC.

The National Comprehensive Cancer Network classified programmed death ligand-1 (PD-L1) as a critical biomarker for predicting patient response to immune checkpoint inhibitors (ICIs) in cancer treatment.^6^^,^^7^ The Food and Drug Administration (FDA) supported the use of tumor mutational burden (TMB; ≥ 10 mutations/mb) as an indicator for successful treatment with pembrolizumab in various cancers, including NSCLC.^8^, ^9^, ^10^

High TMB, PD-L1 expression, and microsatellite instability (MSI) are expected to have strong predictive power in assessing the therapeutic benefits of ICIs.^11^, ^12^, ^13^ Oncogenic drivers and signaling pathway alterations affect PD-L1 levels, TMB, MSI, and the tumor environment, all of which may influence the effect of immunotherapy. In this review, we summarize the oncogenic drivers and signaling pathway alterations that crosstalk with immunotherapy, aiming to highlight individualized and more effective combined immunotherapy for NSCLC.

## Crosstalk between oncogenic drivers and immunotherapy

Oncogenic drivers in NSCLC mainly include Kirsten rat sarcoma viral oncogene homolog (*KRAS*), *EGFR*, V-raf murine sarcoma viral oncogene homolog B (*BRAF*), and human epidermal growth factor receptor 2 (*HER2/erbB2*) mutations, as well as anaplastic lymphoma kinase (*ALK*) and ROS proto-oncogene 1 receptor tyrosine kinase (*ROS1*) rearrangements. Crosstalk between oncogenic drivers and immunotherapy has received much attention.

## *KRAS* mutations

The incidence of *KRAS* mutations in advanced NSCLC patients was approximately 29%–32%.^14^^,^^15^ Patients with NSCLC harboring *KRAS* mutations exhibited higher levels of PD-L1 expression and TMB and increased PD-L1^+^/CD8^+^ tumor-infiltrating lymphocyte (TIL) infiltration than those with the wild-type allele. Compared with the wild-type group, *KRAS*-mutant patients demonstrated superior objective response rates (ORR) to anti-PD-1/PD-L1 immunotherapy.^16^ Retrospective data showed that after anti-PD-1/PD-L1 monotherapy, patients with *KRAS* mutations achieved an ORR of 26%, with a median progression-free survival (mPFS) of 3.2 months and median overall survival (mOS) of 13.5 months. Moreover, PFS was positively associated with PD-L1 expression.^17^ In a real-world therapeutic analysis, *KRAS*-mutant patients exhibited longer PFS with immunotherapy or chemoimmunotherapy (CIT) than wild-type patients, with treatment efficacy positively correlated with PD-L1 expression. However, monotherapy demonstrated inferior outcomes compared to combination regimens (mPFS: 5.6 *vs.* 10.3 months).^18^

A multicenter retrospective study showed that *KRAS* G12 mutations included *KRAS* G12C (47.9%), G12V (20.5%), G12D (17.4%), and G12A (8.2%). After anti-PD-1 alone or anti-PD-1 combined with chemotherapy treatments, the mPFS of *KRAS* G12C, G12D, and G12A isoforms were 4.0–18.0 months, 8.0 months, and 9.0 months, respectively, while the mOS was 15.0 months, 11.5 months, and 20.0 months, respectively.^19^ The LC-SCRUM-Asia study showed that NSCLC patients with *KRAS* G12C had increased immunogenicity (high TMB and high PD-L1 expression). When treated with anti-PD-1/PD-L1, the *KRAS* G12C group obtained better mPFS (3.4 months) than the G12D group (2.0 months).^20^

A Danish nationwide observational register study showed that the OS in the *KRAS* G12C group was similar to that in patients with any *KRAS* mutation and wild-type cases. Survival was positively related to PD-L1 expression regardless of the mutational group.^21^ Retrospective analyses revealed shorter mPFS in *KRAS*-mutant (*KRAS*^Mut^) versus wild-type (*KRAS*^WT^) patients after first-line treatments (4.0 *vs.* 5.1 months) and reduced mOS (12.6 *vs.* 15.4 months). *KRAS* p.G12C mutants benefited from first-line CIT, achieving an mOS of 48.8 months, compared to 24.0 months for non-p.G12C mutants and 22.5 months for *KRAS*^WT^ patients. Second-line immunotherapy improved mOS in *KRAS* p.G12C patients (12.6 months) relative to non-p.G12C (9.4 months) and *KRAS*^WT^ (9.6 months) cohorts.^22^ A systematic review and meta-analysis showed that NSCLC patients with *KRAS* G12C harboring high PD-L1 expression had improved PFS with anti-PD-1 or anti-PD-L1 monotherapy (hazard ratio (HR) = 0.39) compared to other *KRAS* mutations (HR = 0.33).^23^

The *TP53/KRAS* co-mutated subgroup was associated with increased PD-L1 expression and higher TMB and T-cell infiltration. *TP53* or *KRAS* mutation altered DNA replication as well as damage repair-related genes and cell cycle-regulating genes. Analyses of clinical data (MSKCC and KEYNOTE-001) revealed that patients harboring *TP53* or *KRAS* mutations who were treated with pembrolizumab experienced significantly longer PFS than those with wild-type tumors. Importantly, *TP53/KRAS* co-mutated patients demonstrated durable clinical benefit from PD-1 inhibitors.^24^ In a multicenter cohort, NSCLC patients with *KRAS* G12C/*TP53* co-mutations treated with pembrolizumab exhibited superior response rates and PFS. With a median follow-up of 41 months, patients with these co-mutations exhibited significantly prolonged PFS (33.7 months) and OS (65.3 months). These outcomes were associated with increased infiltration of CD4^+^ T cells, CD8^+^ T cells, and B lymphocytes and a proinflammatory tumor microenvironment (TME) driven by robust interferon gamma (IFN-γ) signaling.^25^ A retrospective cohort study further confirmed that patients with *KRAS*^Mut^/*TP53*^Mut^ tumors exhibited prolonged OS when treated with ICI monotherapy or in combination with platinum-based chemotherapy.^26^

Recently, two KRAS G12C inhibitors, sotorasib (AMG 510) and adagrasib (MRTX849), have been sanctioned by the FDA for managing previously treated patients with advanced NSCLC.^27^^,^^28^ Nevertheless, patients with NSCLC harboring concurrent *KRAS* G12C and kelch-like ECH-associated protein 1 (*KEAP1*) mutations appeared to derive less benefit from sotorasib.^29^ *KRAS* mutations were associated with distinct immunological trends, including increased PD-L1 expression and higher TMB. These mutations contributed to an immunosuppressive milieu, marked by the inhibition of T cell infiltration and the recruitment of suppressive cell populations, including myeloid-derived suppressor cells (MDSCs), regulatory T cells (Treg cells), and cancer-associated fibroblasts (CAFs).^30^ Through myelocytomatosis oncogene (MYC) inhibition, KRAS G12C inhibition activated interferon signaling, resulting in the reduction of immunosuppressive cell infiltration and increased infiltration and activation of cytotoxic T cells and antigen presentation.^31^ Patients receiving KRAS inhibitors often develop resistance; however, TKI and ICI combination therapies are promising.^32^^,^^33^

Two trials indicated that newly diagnosed *KRAS* G12C mutant patients with NSCLC had outstanding ORR (49% and 57%) and responded to adagrasib and pembrolizumab combination therapy, which showed a low risk of liver toxicity.^34^ However, the liver toxicity risk of sotorasib,^35^ particularly when administered close to ICI treatment, led to recommendations for a 6-week gap between treatments to reduce the risk.^36^ Adagrasib counteracted tumor immune evasion by increasing the expression of major histocompatibility complex (MHC) class I and reducing the expression of immunosuppressive factors. It also modulated immune cell populations in tumor models, decreasing the percentage of MDSCs while increasing those of M1-polarized macrophages, dendritic cells (DCs), and multiple T-cell subsets. KRAS signaling triggers the release of cytokines that suppress immune responses. However, inhibiting KRAS G12C activated antigen-presenting cells and T cells and improved tumor-intrinsic immune responses.^37^ A real-world retrospective study showed that rare *KRAS* mutations (including G13C, G13D, Q61H, and G12S) benefited from immunotherapy compared with the outcome of chemotherapy (mPFS, 7.3 *vs.* 3.4 months; mOS, 13.3 *vs.* 5.2 months).^38^

Advanced NSCLC patients with *KRAS* mutations benefited from ICIs, especially the *KRAS* G12C, *KRAS G12C/TP53* co-mutations cohort, and the rare *KRAS* mutation group.^19^^,^^20^^,^^22^^,^^23^^,^^25^^,^^38^ The combination of adagrasib and pembrolizumab was also a promising treatment for patients with the *KRAS* G12C isoform.^34^

## *EGFR* mutations

*EGFR* mutations were present in approximately 26.4% of NSCLC cases.^39^ EGFR-TKIs were favored as the initial treatment for patients with major *EGFR* mutations, such as exon 19 deletions (Ex19del) and the exon 21 L858R substitution (L858R).^6^ A retrospective study reported that *EGFR*-mutant patients exhibited an ORR of 12%, mPFS of 2.1 months, and mOS of 10 months after anti-PD-1/PD-L1 mono-immunotherapy. PFS was positively associated with PD-L1 expression.^17^ The real-world therapeutic analysis showed that patients with *EGFR*-mutant NSCLC exhibited low PD-L1 expression levels, while the mPFS was only 3.0 months after treatment with first-line immunotherapy.^18^ Mutations in *EGFR* increased the secretion of PD-L1-positive small extracellular vesicles (sEVs). PD-L1-positive sEVs were negatively related to the presence of CD8^+^ T cells in the TME. This may be a reason for the limited efficacy of immunotherapy in *EGFR*-mutant patients.^40^ In *EGFR*-mutated NSCLC, transforming growth factor beta (TGF-β) was upregulated by EGFR activation and subsequent extracellular signal-regulated kinase 1/2-p90RSK (ERK1/2-p90RSK) phosphorylation. The infiltration, proliferation, and cytotoxicity of CD8^+^ T cells may be inhibited by TGF-β directly. This may serve as a resistance mechanism to immunotherapy in *EGFR*-mutated NSCLC.^41^

A retrospective analysis indicated that among patients with *EGFR*-mutant NSCLC who received ICI monotherapy following EGFR-TKI failure, patients harboring the *EGFR* L858R mutation achieved significantly longer survival than those with Ex19del (HR = 0.35).^42^ In contrast, another study evaluated patients with *EGFR*-mutant NSCLC who were treated with either ICI monotherapy or ICI-based combination therapy after EGFR-TKI resistance and reported that patients with the L858R mutation exhibited significantly shorter mPFS (mPFS: 2.5 *vs.* 6.7 months, HR = 1.80) and mOS (mOS: 9.8 *vs.* 26.9 months, HR = 2.48) than Ex19del carriers.^43^ This apparent contradiction likely stems from differences in treatment regimens: the survival advantage for Ex19del was specifically observed in the context of combination therapy, suggesting that the addition of chemotherapy or other agents may fundamentally alter the immunobiology of this subtype, enhancing its response to immunotherapy. In contrast, the L858R subtype may show distinct sensitivity patterns to ICI monotherapy. These findings imply that the optimal immunotherapy strategy depends on both the *EGFR* mutation subtype and the treatment modality. While outcomes with ICI monotherapy remain inconsistent, Ex19del-mutant tumors appear to derive marked benefit from ICI-based combinations after TKI failure, underscoring the need for biomarker-driven studies to guide personalized treatment selection.

*EGFR* uncommon mutations included G719X, L861Q, S768I, and exon 20 insertions. NSCLC patients with uncommon *EGFR* mutations showed high rates of concomitant PD-L1 expression and CD8^+^ TIL infiltration in the tumor microenvironment. This may indicate a good response to PD-1 blockade in patients with rare *EGFR* mutations.^44^ A retrospective analysis showed that patients with rare *EGFR* mutations (exon 20 insertions or G719X) who received ICI treatment exhibited a longer PFS and a better ORR than patients with major *EGFR* mutations (L858R or 19Del).^45^After the failure of EGFR-TKI treatments, NSCLC patients with uncommon *EGFR* mutations (G719X or exon 20 insertions) demonstrated a better response to ICIs, showing an ORR of 71%, compared with the ORR of 35.7% in *EGFR* common mutations. Patients without T790M mutations also exhibited a significantly longer mPFS.^46^

Patients with *EGFR* exon 20 mutations in NSCLC were associated with a better response (50%) to ICI treatment and had a significantly better disease control rate (67%).^47^ However, compared to the wild-type, in the TME of *EGFR* ex20ins tumors, there were significantly lower levels of CD8^+^ Forkhead box P3^-^ (FOXP3^-^) T cells and antitumor cytokines produced by M1-like macrophages but increased numbers of M2-like macrophages. This suggests that ICI-combination therapy may be a more appropriate treatment option compared to ICI monotherapy.^48^

Following the initial failure of EGFR-TKI therapy, patients with a shorter PFS tended to experience better efficacy with subsequent immunotherapy.^49^ Patients with short TKI-PFS demonstrated increased CD8^+^ effector cells and lower ratios of M2 to M1-like macrophages. Patients with short TKI-PFS showed longer PFS (HR = 0.51) and OS (HR = 0.48) and better ORR (33.3 *vs.* 10.0%) from combined immunotherapy compared with traditional chemotherapy.^50^ Patients with positive p53 expression exhibited a better response to ICI-based therapy after osimertinib (EGFR-TKI) treatment, achieving an ORR of 26.3%, mPFS of 4.0 months, and mOS of 7.4 months. Conversely, high AXL (a receptor tyrosine kinase) expression negatively impacted ICI-based therapy outcomes; patients with low AXL levels had an ORR of 17.2%, mPFS of 3.2 months, and mOS of 8.6 months.^51^ Advanced NSCLC patients with *EGFR* without T790M mutations and high PD-L1 expression may benefit from ICI-based treatment compared to chemotherapy after prior EGFR-TKI treatment failure. ICI combined with chemotherapy appeared to be the preferred treatment (mPFS, 10.3 *vs.* 7.0 months; mOS, 41.6 *vs.* 32.4 months) compared with ICI mono-immunotherapy.^52^

In NSCLC, activated EGFR signaling suppresses the immune response through various mechanisms, including upregulation of PD-L1 levels in cancer cells, which promotes T-cell death and recruits tumor-associated macrophages and Treg cells that produce suppressive cytokines and metabolites. In contrast, EGFR-TKIs enhance CD8^+^ T cells and DCs activity, markedly decrease the number of FOXP3^+^ Treg cells, prevent M2 macrophage polarization, downregulate tumor PD-L1 expression, and increase MHC class I and II antigen presentation.^53^ Another study suggested that EGFR-TKI therapy in patients with *EGFR*-mutant NSCLC and high PD-L1 did not alter CD8^+^ TILs; instead, it increased CD73 in tumor cells, potentially elevating TMB. These trends were suggestive of alterations to the TME that are beneficial for further immunotherapy or combined treatment strategies.^54^

Combining ICIs with targeted therapies poses significant toxicity management challenges in clinical practice. For instance, pembrolizumab combined with gefitinib resulted in grade 3/4 liver toxicity in five of seven patients, making it an impractical treatment option. Pembrolizumab plus erlotinib (EGFR-TKI) was a viable combination, with adverse events similar to those expected for monotherapy; the most common treatment-related adverse events were rash (50.0%), acneiform dermatitis, diarrhea, hypothyroidism, and pruritus. However, this combination failed to improve the ORR compared to monotherapy, warranting further evaluation of its potential effects on other efficacy outcomes.^55^ Finally, the combination of erlotinib and ipilimumab (an anti-cytotoxic T lymphocyte antigen 4 [CTLA4] monoclonal antibody) exhibited significant efficacy in patients with advanced NSCLC harboring *EGFR* mutations; however, the trial was terminated owing to grade 3 colitis.^56^ The KEYNOTE-021 study indicated that combining pembrolizumab with ipilimumab enhanced efficacy in patients with advanced NSCLC harboring *EGFR* or *ALK* aberrations after chemotherapy or targeted therapy failure, albeit this regimen was associated with significant toxicity.^57^

These findings underscore several practical considerations for clinicians and trial designers: (1) Patient selection: Combine ICIs and EGFR-TKIs with extreme caution, especially in patients with preexisting liver conditions, abnormal liver function, or a history of colitis. (2) Toxicity monitoring: Implement rigorous monitoring of liver function and diarrhea symptoms, with established protocols for early intervention. (3) Alternative strategies: Given the toxicity-efficacy trade-off, sequential therapy (TKI followed by ICI) may be a more viable option for patients with targetable mutations. Further research is needed on the time interval of sequential treatment. (4) Need for novel approaches: Future research should focus on optimized dosing schedules and biomarkers predictive of efficacy and toxicity to improve the therapeutic window.

EGFR signaling regulates CD73 expression. In a xenograft model of *EGFR*-mutated NSCLC, combining anti-PD-L1 and anti-CD73 antibodies significantly reduced tumor growth. This treatment increased gene expression related to inflammation and T-cell function, boosted the number of tumor-infiltrating CD8^+^ T cells, and enhanced the production of IFN-γ and tumor necrosis factor-α (TNF-α).^58^

Patients with NSCLC harboring *EGFR* mutations exhibit a limited response to mono-immunotherapy, rendering EGFR-TKIs the preferred first-line treatment. Notably, patients with uncommon *EGFR* mutations, such as G719X, L861Q, S768I, and exon 20 insertions, may display a favorable response to PD-1 blockade.^44^ For patients with disease progression following EGFR-TKI failure, those harboring G719X mutations, exon 20 insertions, positive p53 expression, or low AXL expression tend to achieve superior responses to ICI therapy.^46^^,^^51^ Additionally, patients with advanced *EGFR*-mutant NSCLC who lack the T790M mutation or harbor an Ex19del mutation may derive clinical benefits from ICI-based combination therapy following disease progression on prior EGFR-TKI treatment.^43^^,^^52^ *In vivo*, combined treatment with anti-PD-L1 and anti-CD73 antibodies exerts potent antitumor efficacy.^58^ However, clinically, the combination of EGFR-TKIs and immunotherapy is associated with significant adverse events, including hepatotoxicity and severe gastrointestinal reactions, limiting its widespread application.

## *BRAF* mutations

Mutations in the *BRAF* gene were present in approximately 2%–4% of NSCLC cases.^59^ BRAF, a constituent of the RAF family of kinases, plays a crucial role in the mitogen-activated protein kinase (MAPK) pathway, influencing cell growth, differentiation, and survival. *BRAF* mutations have been categorized into V600 (class 1) and non-V600 (classes 2 and 3) types.^60^^,^^61^

BRAF inhibitors, including sorafenib, dabrafenib, vemurafenib, and BGB-283, have demonstrated efficacy against *BRAF*-mutant NSCLC.^62^^,^^63^ Studies have indicated that combining dabrafenib (BRAF-TKI) and trametinib (mesenchymal to epithelial transition factor [MET] inhibitor) is an effective approach for treating NSCLC with *BRAF*^*V600E*^ mutations.^64^, ^65^, ^66^, ^67^ Similarly, encorafenib (BRAF-TKI) and binimetinib (MET inhibitor) have demonstrated both efficacy and tolerability in *BRAF*^*V600E*^-mutated metastatic NSCLC.^68^

A retrospective study showed that patients with *BRAF* mutations and advanced NSCLC exhibited an ORR of 24%, mPFS of 3.1 months, and mOS of 13.6 months after receiving mono-immunotherapy. PFS was positively associated with smoking status.^17^ After anti-PD-1/PD-L1 immunotherapy, the response rate was 28.6%, and the mPFS was 2.2 months in *BRAF*-mutant patients with advanced NSCLC.^69^ The *BRAF*-mutant cohort showed similar ratios of CD8^+^ T cell and Treg infiltration compared with wild-type NSCLC. There was no significant difference in immune-related signaling pathways associated with antigen processing and presentation, interleukin-2 (IL-2)-STAT5 signaling, IL-6-JAK-STAT3 signaling, or the inflammatory response. These TME features in the V600E group were similar to those observed in the non-V600E NSCLC cohort. A retrospective study confirmed similar OS (HR = 0.85) and PFS (HR = 1.02) in patients with *BRAF* mutations and wild type after treatment with ICI monotherapy or combined therapies. Similar results were observed in response to immunotherapy in *BRAF*^*V600E*^ or *BRAF*^*non-V600E*^ NSCLC.^70^ In the two clinical cohorts, patients with advanced NSCLC were treated with an anti-PD-1/PD-L1 single agent. In the MDACC and CGDB cohorts, the *BRAF*-mutant group exhibited superior outcomes, with the highest ORR, PFS, and OS. Specifically, in the CGDB cohort, PFS was 9.8 months for V600E and 5.4 months for non-V600E mutations, with corresponding OS values of 20.8 and 14.9 months, respectively.^71^ The IMAD2 study reported an ORR of 26%, mPFS of 5.3 months, and mOS of 22.5 months in V600E-mutant patients, while non-V600E mutations showed an ORR of 35%, mPFS of 4.9 months, and mOS of 12 months.^72^ Real-world evidence supported the effectiveness of ICI-based treatments, with ORRs of 38% for V600E and 43% for non-V600E mutations, mPFS of 10.5 months for V600E and 10.8 months for non-V600E.^67^ Retrospective analyses have indicated that ICI combined with chemotherapy improves mPFS in patients with advanced NSCLC harboring *BRAF* mutations, with an mPFS of 12.6 months overall and 17.5 months in those with *BRAF*^*V600E*^ mutations.^73^^,^^74^ The NADIM cohort demonstrated a 100% pathological complete response rate in *BRAF*-mutant patients undergoing neoadjuvant CIT, compared to 44.3% in wild-type patients.^75^ A Chinese expert consensus recommends ICIs as first-line treatment for *BRAF*^*non-V600E*^ mutations and ICI combinations for *BRAF*^*V600E*^ mutations when TKI therapy is inaccessible or resistance occurs.^76^

## *ALK* rearrangements

The incidence of *ALK* rearrangements in advanced NSCLC patients was approximately 4.1%–5%.^14^^,^^15^ A retrospective study showed that patients with *ALK* rearrangements exhibited the worst ORR of 0%, mPFS of 2.5 months, and mOS of 17 months after receiving anti-PD-1/PD-L1 mono-immunotherapy.^17^ Patients with *ALK*-positive NSCLC treated with nivolumab (PD-1 inhibitor) or pembrolizumab exhibited a PFS of 0.6 months, despite high levels of PD-L1 expression.^77^ Patients with *ALK* rearrangements displayed decreased levels of CD3^+^ and CD8^+^ T cells and CD20^+^ B cells, accompanied by an increase in CD4 helper T cells. Additionally, the expression of immune checkpoints, such as T-cell immunoglobulin and mucin-domain containing protein 3 (Tim-3), CTLA4, lymphocyte activation gene (LAG), and PD-1, in CD8^+^ T cells was lower in *ALK*-positive NSCLC than in NSCLC with *EGFR/KRAS* mutations.^78^

Phase III trials of various ALK inhibitors have demonstrated potential benefits for patients with *ALK*-rearranged NSCLC.^79^ However, resistance to ALK*-*TKIs in these patients may arise from both on-target and off-target mechanisms, with the latter involving alternative signaling pathways or phenotypic transformations.^80^

An open-label, multicenter, phase 1B study showed that the combination of ceritinib (ALK-TKI) and nivolumab appeared promising in the treatment of advanced NSCLC with *ALK* rearrangements. Among ALK TKI-naive patients, the ORR was 83% in the 450 mg ceritinib cohort and 60% in the 300 mg ceritinib cohort. In ALK-TKI-pretreated patients, the ORR was 50% in the 450 mg ceritinib cohort and 25% in the 300 mg ceritinib cohort. The therapeutic effect was positively correlated with PD-L1 levels. However, this combination therapy increased the risk of developing a rash compared to monotherapy.^81^ Notably, a complete response for 16 months had been reported in a patient treated with nivolumab following ceritinib and platinum-based chemotherapy.^82^ A case study highlighted the combination of atezolizumab (PD-L1 inhibitor) with bevacizumab (vascular endothelial growth factor monoclonal antibody) and chemotherapy following the failed treatment of three generations of ALK-TKIs.^83^

Moreover, combining crizotinib (ALK-TKI) with nivolumab was not recommended, given reports of severe liver toxicity and fatalities.^84^ Similarly, combining crizotinib with pembrolizumab could lead to significant liver damage and fatigue in patients, leading to treatment discontinuation, as reported in a previous study.^85^

A phase I/II study demonstrated that alectinib (second-generation ALK-TKI) combined with bevacizumab was well tolerated in advanced ALK-rearranged NSCLC, with objective responses in 100% of ALK-TKI-naive and 60% of pretreated patients (mPFS: NR and 9.5 months, respectively).^86^

While immunotherapy alone is ineffective in ALK-rearranged NSCLC, combination therapy with ALK-TKIs and ICIs shows promise, particularly in TKI-naive patients. Future research should focus on novel inhibitor combinations and address drug toxicities to enhance treatment outcomes.

## *ROS1* rearrangements

The incidence of *ROS1* rearrangements in patients with NSCLC was approximately 1–3%.^15^^,^^87^ The upregulation of PD-L1, regulated by the ROS1-SHP2 signaling pathway, and the increase in CD3^+^PD-1^+^ T cells suggested that *ROS1* rearrangements in NSCLC may display a good clinical response to anti-PD-L1 therapy.^88^ A retrospective study showed that patients with *ROS1* rearrangements exhibited an ORR of 17% after receiving anti-PD-1/PD-L1 mono-immunotherapy.^17^

Most patients with *ROS1* rearrangements in NSCLC had low levels of PD-L1 and TMB. Patients with *ROS1* rearrangements displayed an ORR of 13% following anti-PD-1/PD-L1 treatment. Patients treated with chemo- and anti-PD-1 antibodies achieved an ORR of 83%. Consequently, combining chemotherapy with immunotherapy could be more efficacious.^87^ A retrospective study indicated that patients with *ROS1*-rearranged NSCLCs benefited from ICI combined with chemotherapy, including patients with brain metastasis.^89^ Another retrospective study showed that the mPFS of patients with *ROS1* rearrangements was 15.2 months (mPFS 3.9 months in the chemotherapy group) after treatment with ICI plus chemotherapy.^74^ After failed TKI therapy, a combination of atezolizumab (PD-L1 inhibitor) with or without bevacizumab and platinum-pemetrexed was shown to be a safe and effective treatment option for patients with NSCLC and *ALK/ROS1* rearrangements.^90^

Among patients with NSCLC and *ROS1* rearrangements, the response to immunotherapy was generally moderate. Chemotherapy combined with immunotherapy may be a promising treatment for these patients.

## *HER2* mutations

*HER2 (ERBB2)* mutations occurred in 5.6% of the Chinese and 5.2% of the US NSCLC cohorts, with low TMB characterizing HER2 exon 20 NSCLC.^91^ While anti-PD-1/PD-L1 monotherapy demonstrated limited efficacy in advanced NSCLC with *HER2* mutations, with PFS showing positive correlation with smoking history,^17^ it exhibited more promising activity in *HER2*-mutant NSCLC, achieving a 29% ORR and 57% disease control rate.^47^ The IMAD2 study indicated that the response rate was 27%, the mPFS was 2.2 months, and the mOS was 20.4 months in patients with *HER2* mutations after treatment with anti-PD-1 or anti-PD-L1.^72^

Combining ICIs with platinum-based chemotherapy as first-line treatment may improve PFS in patients with *HER2*-mutant NSCLC, possibly related to high TMB.^92^ A Chinese retrospective analysis reported an ORR of 51.6%, mPFS of 8.2 months, and mOS of 21.0 months with 1 L-ICI plus chemotherapy, while 2/3 L-ICI plus chemotherapy showed an mPFS of 5.4 months and mOS of 16.2 months.^93^ However, another study found that HER2 exon 20 insertions were associated with a worse mPFS of 8.2 months under ICI plus chemotherapy (HR = 2.39) (Table 1).^74^

**Table 1:** Impact of oncogenic drivers on ICI efficacy in NSCLC.

Oncogene	Therapy regimen	ORR	mPFS, months (95% CI)	mOS, months (95% CI)	Reference
*KRAS*	anti-PD-1/PD-L1	OR = 1.51; (1.17–1.96; *p* = 0.002)	NA	NA	16
	anti-PD-1/PD-L1	26%	3.2 (2.7–4.5)	13.5 (9.4–15.6)	17
	anti-PD-1/PD-L1 or anti-PD-1/PD-L1 + chemo	NA	anti-PD-1/PD-L1 *vs.* anti-PD-1/PD-L1+ chemo, (5.6 *vs.* 10.3, *p* < 0.01)	NA	18
	anti-PD-1 or anti-PD-1 + chemo	NA	G12C: 4–18, G12D: 8, G12A: 9, G12V: 3–12	G12C: 15, G12D: 11.5, G12A: 20.0, G12V: 9	19
	anti-PD-1/PD-L1	NA	G12C: 3.4, G12D: 2.0, G12V: 4.2	NA	20
	1/2L^a^	NA	NA	anti-PD-1/PD-L1 + chemo (1L^b^): p.G12C: 48.8 anti-PD-1/PD-L1 (2L^c^): p.G12C: 12.6	22
	anti-PD-1/PD-L1	NA	G12C: HR: 0.39 (0.25–0.63)	NA	23
	Adagrasib + anti-PD-1	G12C*:* Trial 1: 49%, Trial 2: 57%	NA	NA	34
	ICI-based treatment	NA	Rare *KRAS*: ICI-based treatment *vs.* chemo: 7.3 *vs.* 3.4, *p* < 0.001	Rare *KRAS*: ICI-based treatment *vs.* chemo: 13.3 *vs.* 5.2, *p* < 0.001	38
*KRAS* or *TP53*	anti-PD-1	NA	*TP53*^*Mut*^: 14.5, *KRAS*^*Mut*^: 14.7, WT: 3.5, *p* = 0.012	NA	24
*KRAS* and *G12C*/*TP53*	anti-PD-1	RR: G12C/TP53 comut: 69.7%, non-G12C/TP53 comut: 55.4%	PFS: G12C*/TP53* comut: HR = 0.59, *p* = 0.009 non-G12C*/TP53* comut: HR = 0.7, *p* = 0.047	OS: G12C*/TP53* comut: HR = 0.72, *p* = 0.16 non-G12C*/TP53* comut: HR = 0.56, *p* = 0.002	25
	ICI monotherapy or ICI + platinum-based chemotherapy	NA	NA	OS univariate: HR = 0.56 (0.38–0.84), *p* = 0.0044 OS multivariate: HR = 0.53 (0.35–0.79), *p* = 0.0021	26
*EGFR*	anti-PD-1/PD-L1	12%	2.1 (1.8–2.7)	10 (6.7–14.2)	17
	1L^b^ anti-PD-1/PD-L1	NA	3	NA	18
	EGFR-TKI + anti-PD-1	Uncommon: 71% Common: 35.7%	NA	NA	45
	EGFR-TKI + anti-PD-1/PD-L1 or anti-PD-1/PD-L1 + chemo	*TP53*^*+*^: 26.3% low AXL: 17.2%	*TP53*^*+*^: 4.0 low AXL: 3.2	*TP53*^*+*^: 7.4 low AXL: 8.6	^51^
	anti-PD-1/PD-L1	*EGFR* exon 20: 50%	NA	NA	^47^
*EGFR* with non-T790M	ICI or ICI + chemo	NA	ICI: 7 ICI + chemo: 10.3	ICI: 32.4 ICI + chemo: 41.6	^52^
*BRAF*	anti-PD-1/PD-L1	24%	3.1 (1.8–4.6)	13.6 (7.4–22.5)	17
	anti-PD-1/PD-L1	28.6%	2.2 (0.9–NA)	NA	^69^
	anti-PD-1/PD-L1 or anti-PD-1/PD-L1 combined therapy	NA	PBS: HR: 1.02 (0.72–1.44); *p* = 0.91	OS: HR: 0.85 (0.56–1.30), *p* = 0.47	^70^
	anti-PD-1/PD-L1	MDACC: 62%	PBS: MDACC: 7.4 CGDB: V600E: 9.8, non-V600E: 5.4	OS: MDACC: 35.6 CGDB:600E: 20.8, non-V600E: 14.9	^71^
	anti-PD-1/PD-L1	V600E: 26% non-V600E: 35%	V600E: 5.3 (2.1–NR) non-V600E: 4.9 (2.3-NR)	V600E: 22.5 (8.3–NR) non-V600E: 12 (6.8–NR)	^72^
	ICI-based therapy	V600E: 38% non-V600E: 43%	V600E: 10.5 non-V600E: 10.8	NA	^67^
	ICI + chemo	44%	12.6 (5.8–NA)	NA	^73^
	ICI + chemo	NA	V600E: 17.5	NA	^74^
	anti-PD-1 + chemo	pCR: 100%	NA	NA	^75^
*ALK*	anti-PD-1/PD-L1	0%	2.5 (1.5–3.7)	17	17
	anti-PD-1	NA	0.6 (0.2–2.1)	NA	^76^
	Ceritinib + anti-PD-1	TKI-naive:	NA	NA	^81^
		450 mg Cer: 83% (35.9%–99.6%)			
		300 mg Cer: 60% (26.2%–87.8%)			
		TKI-pretreated:			
		450 mg Cer: 50% (15.7%–84.3%)			
		300 mg Cer: 25% (5.5%–57.2%)			
*ROS1*	anti-PD-1/PD-L1	17%	NA	NA	17
	anti-PD-1/PD-L1 or anti-PD-1 + chemo	anti-PD-1/PD-L1: 13% (2%–38%)	NA	NA	^87^
		anti-PD-1 + chemo: 83% (36%–100%)			
	ICI + chemo	NA	15.2	NA	^74^
*HER2*	anti-PD-1/PD-L1	7%	2.5 (1.8–3.5)	20.3	17
	anti-PD-1/PD-L1	27%	2.2 (1.7–15.2)	20.4 (9.3–NR)	^72^
	anti-PD-1/PD-L1	29%	NA	NA	^47^
	ICI + chemo	1 L: 51.6%	1L^b^: 8.2 (6.4–10)	1L^b^: 21.0 (12.2-29.8)	^93^
			2/3L^d^: 5.4 (4.4–6.4)	2/3L^d^: 16.2 (1.1–31.3)	
	ICI + chemo	NA	*HER2* exon 20 insertions: 8.2, HR: 2.39 (1.19–4.77, *p* = 0.014)	NA	^74^

Note: ALK, anaplastic lymphoma kinase; AXL: a receptor tyrosine kinase; BRAF, V-raf murine sarcoma viral oncogene homolog B; Cer, Ceritinib; chemo, chemotherapy; comut, co-mutation; EGFR, epidermal growth factor receptor; HER2, human growth factor receptor 2; HR, hazard ratio; ICI, immune checkpoint inhibitor; KRAS, Kirsten rat sarcoma viral oncogene homolog; mOS, median overall survival; mPFS, median progression-free survival; NR, not reached; NSCLC: non-small cell lung cancer; OR, odds ratio; ORR, overall response rate; pCR, pathological complete response rate; PD-1, programmed death-1; PD-L1, programmed death ligand-1; ROS1, ROS proto-oncogene 1 receptor tyrosine kinase; RR, response rate; TKI, tyrosine kinase inhibitor; WT, wild-type.

1/2L^a^, first or second line; 1L^b^, first line; 2L^c^, second line; 2/3L^d^, second or third line.

Thus, patients with *HER2*-mutant NSCLC may benefit from mono-immunotherapy, particularly CIT, but those with HER2 exon 20 insertions might face worse outcomes with CIT.

## Types of gene mutations in NSCLC resistant to immunotherapy

### *KRAS* G12D and *KRAS* G12V mutations

A retrospective study compared *KRAS* G12C with G12D and G12A isoforms. The G12V mutation had the shortest mPFS (4.0 months) and mOS (9.0 months) under ICI-alone or ICI-chemotherapy combination, while the global mPFS was 6.0 months and global mOS was 15.0 months, likely due to a higher proportion of older patients and lower PD-L1 expression.^19^ The *KRAS* G12D mutation suppressed PD-L1 expression via the P70S6K/phosphoinositide 3-kinase/protein kinase B (PI3K/AKT) pathway. It also decreased chemokine ligand 10 (CXCL10) and CXCL11 levels by downregulating the expression of high-mobility group protein A2, leading to a decreased proportion of CD8^+^ T cells. Consequently, ICIs exhibited limited efficacy in patients with *KRAS*-G12D-mutant NSCLC, suggesting that CIT may offer improved efficacy.^94^

### *STK11 (LKB1), KEAP1, SMARCA4,* and PBRM1 mutations

Compared with wild-type NSCLC, non-squamous NSCLC patients with serine/threonine kinase 11 (*STK11*) mutations had significantly reduced outcomes in CIT: lower ORR (25.1% *vs.* 40.5%), mPFS (3.9 *vs.* 6.3 months), and mOS (10.4 *vs.* 15.2 months). Similarly, *KEAP1*-mutated patients showed impaired CIT outcomes (ORR: 23.3% *vs.* 41.1%; mPFS: 3.3 *vs.* 6.7 months; mOS: 8.1 *vs.* 16.9 months). *SMARCA4* mutations were also associated with worse CIT outcomes (ORR: 21.9% *vs.* 39.1%; mPFS: 2.7 *vs.* 6.1 months; mOS: 8.1 *vs.* 15.0 months). The *KRAS*-mutant subgroup with concurrent *STK11*, *KEAP1*, or *SMARCA4* mutations had the worst CIT outcomes.^95^ Another study found that NSCLC patients with *KRAS* mutations and PD-L1-negative expression had poorer OS after frontline CIT, related to *STK11/KEAP1* co-mutations.^96^

In NSCLC, the cold TME associated with different gene mutations was linked to poor ICI efficacy. In the Memorial Sloan Kettering Cancer Center/Rome and DFCI cohorts, lung adenocarcinoma (LUAD) patients with *KEAP1* mutations (clonal with loss of heterozygosity, *KEAP1* C-LOH) had shorter PFS and OS than *KEAP1* wild-type cases, with fewer total CD8^+^ T cells, lower total PD-1^+^ immune cells (including CD8^+^ PD-1^+^ T cells), and lower PD-L1 expression in *KEAP1* C-LOH tumors (no FOXP3^+^ T-cell difference).^97^ Patients with *KEAP1* mutations co-mutated with polybromo 1(*PBRM1*), *SMARCA4*, and *STK11* had higher TMB but had reduced immunotherapy efficacy compared to patients with single-mutant and wild-type, with lower scores in IFN-γ response, lymphocyte infiltration, macrophage signatures, T-cell receptor (TCR) richness and diversity, Th1 gene expression, and immunomodulatory gene expression.^98^

*PBRM1* mutations in NSCLC were associated with significantly shorter PFS and OS under immune checkpoint blockade (ICB) therapy. The cold TME, characterized by lower macrophage levels, reduced IFN-γ response, CD8^+^ T cells, and activated CD4^+^ memory T cells, as well as poorer TCR richness and diversity, contributed to ICI resistance.^99^ In *murine models of liver kinase B1* (*LKB1*)-deficient NSCLC, elevated ELR^+^ CXC chemokines induced granulocytic myeloid-derived suppressor cell (G-MDSC) amplification and suppressed the TME. Combining all-trans-retinoic acid with anti-PD-1 therapy could improve T-cell proliferation by inhibiting G-MDSCs and potentially overcome ICI resistance.^100^

In a randomized phase III POSEIDON trial, patients with NSCLC who had mutations in *STK11* and/or *KEAP1* derived clinical benefit from chemotherapy combined with dual ICB (durvalumab, PD-L1 inhibitor, and tremelimumab, CTLA4 inhibitor). Loss of *KEAP1* was the strongest genomic predictor for this combination therapy. Dual ICB reprogrammed the myeloid cell compartment toward inducible nitric oxide synthase-expressing phenotypes and engaged CD4^+^ effector cells. The use of chemo-immunotherapy with dual ICB was recommended for patients with NSCLC who had *STK11* and/or *KEAP1* alterations.^101^

A meta-analysis reported that *STK11*^Mut^ *TP53*^Mut^ patients showed a trend toward improved PFS compared to *STK11*^Mut^ *TP53*^WT^ NSCLC patients treated with ICIs. In the DFCI cohort, patients with *STK11* ^Mut^ *TP53* ^Mut^ had a higher ORR (42.9% *vs.* 16.7%) and a longer time to treatment failure (14.5 *vs.* 4.5 months). The TME characteristics of the *STK11*^Mut^ *TP53* ^Mut^ subtype included higher TMB and tumor neoantigen load (TNB) and greater CD8^+^ T-cell and natural killer (NK) cell numbers. A pronounced cyclic GMP-AMP (cGAMP) synthase (cGAS) (STING) signature and increased expression of genes related to glycolysis/glutamine metabolism (such as *MYC* and *HIF-1A*) were also obvious features of *STK11*^Mut^ *TP53*^Mut^ tumors.^102^ To overcome primary resistance to ICIs in NSCLC with *STK11* mutation, metformin in combination with pembrolizumab could effectively increase the infiltration of CD8^+^ T cells and inhibit the K48-linked ubiquitination of stimulator of interferon genes.^103^

In summary, patients with *KRAS* G12V mutations might exhibit the worst response to ICIs alone or ICIs combined with chemotherapy.^19^ The *KRAS*-G12D cohort showed poor results after ICI treatments but may exhibit a better outcome from CIT.^94^ Patients with *KEAP1* C-LOH displayed shorter PFS and OS after immunotherapy.^97^ Individuals carrying *PBRM1* mutations and *LKB1* defects were also associated with poor ICB therapy.^99^^,^^100^ CIT demonstrated poor efficacy in patients with single mutations in *STK11*, *KEAP1*, or *SMARCA4* compared with the wild type. Compared with single mutation and wild-type individuals, the cohort carrying *KRAS* co-mutations with *STK11*, *KEAP1*, or *SMARCA4* had a worse prognosis for CIT.^95^

**Figure 1:**
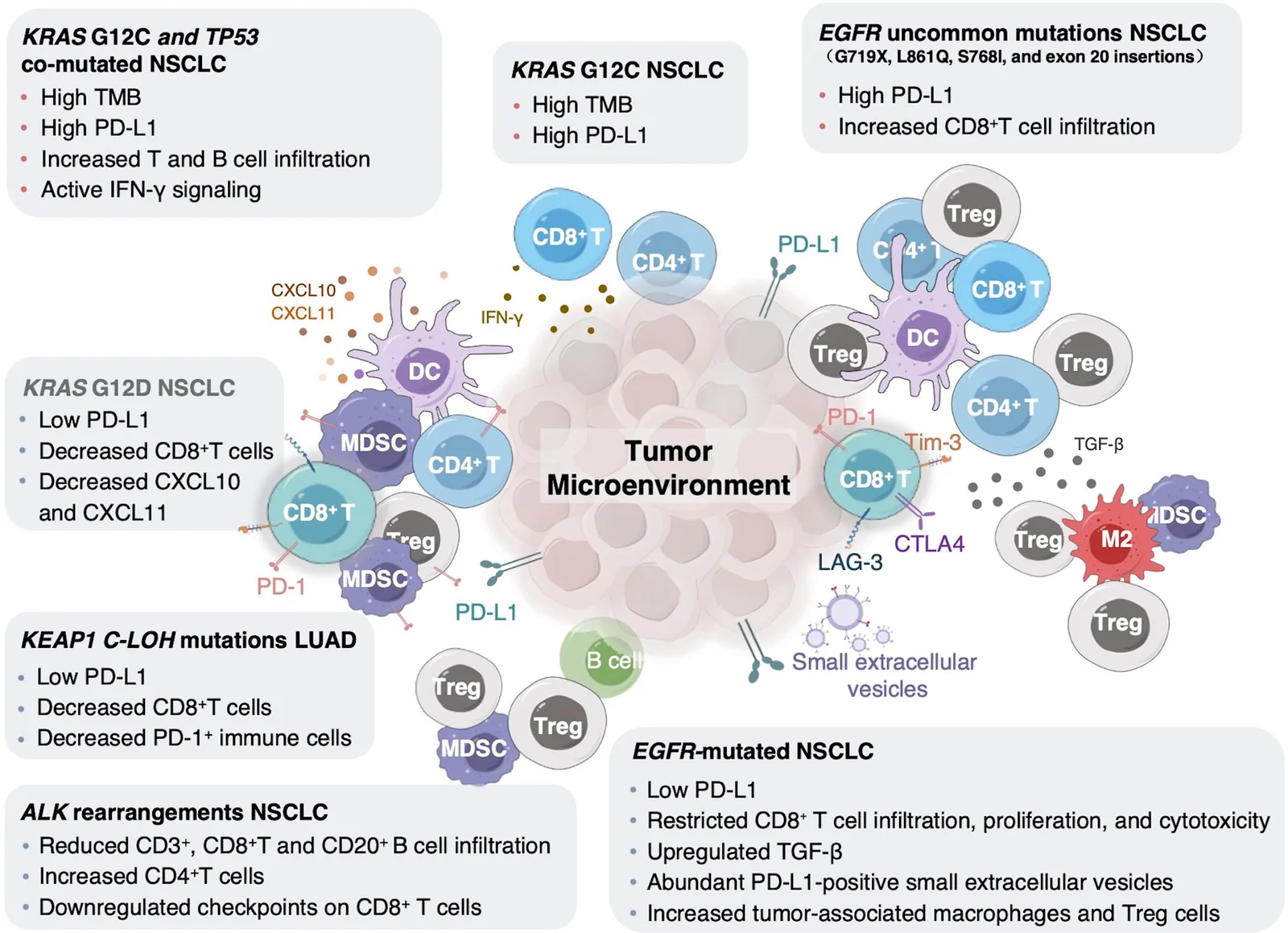
Tumor microenvironment features of distinct oncogenic mutation subtypes in NSCLC. *KRAS G12C-*mutant NSCLC exhibits high tumor mutational burden (TMB) and high PD-L1 expression. In addition to elevated PD-L1 expression and high TMB, *KRAS* G12C/TP53 co-mutated NSCLC is associated with increased infiltration of CD4^+^ T cells, CD8^+^ T cells, and B lymphocytes, as well as a pro-inflammatory TME driven by robust IFN-γ signaling. Patients with NSCLC harboring uncommon *EGFR* mutations (*e.g.*, G719X, L861Q, S768I, and exon 20 insertions) exhibit high levels of PD-L1 expression and CD8^+^ T cell infiltration in the TME. The *KRAS* G12D mutation downregulates PD-L1, CXCL10, and CXCL11 expression levels, which in turn lead to decreased CD8^+^ T cell infiltration. Patients with *EGFR*-mutant NSCLC exhibit low PD-L1 expression levels; notably, EGFR activation upregulates PD-L1-positive small extracellular vesicles and TGF-β, potentially inhibiting the infiltration, proliferation, and cytotoxicity of CD8^+^ T cells. Patients with LUAD harboring *KEAP1* mutations (*e.g.*, clonal with loss of heterozygosity, *KEAP1* C-LOH) possess a low number of total CD8^+^ T cells, low levels of PD-1^+^ immune cells, and reduced PD-L1 expression. Patients with *ALK* rearrangements display decreased numbers of CD3^+^ T, CD8^+^ T, and CD20^+^ B cells, accompanied by increased CD4^+^ T cell infiltration. Additionally, CD8^+^ T cells exhibit low expression levels of immune checkpoints, including Tim-3, CTLA4, LAG-3, and PD-1. Note: CTLA4, cytotoxic T lymphocyte antigen 4; CXCL10, chemokine ligand 10; CXCL11, chemokine ligand 11; IFN-γ, interferon-γ; LAG-3, lymphocyte activation gene 3; LUAD, lung adenocarcinoma; NSCLC, non-small cell lung cancer; PD-1, programmed cell death protein 1; PD-L1, programmed death ligand 1; TGF-β, transforming growth factor beta;Tim-3, T-cell immunoglobulin and mucin-domain containing protein 3; TMB, tumor mutational burden; TME, tumor microenvironment.

Distinct NSCLC oncogenic mutation subtypes exhibit divergent TME characteristics, contributing to the heterogeneity in immunotherapy responses across different genetic alteration types. Some mutations are associated with sensitive subtypes, promoting immune responses and enhancing ICI sensitivity. These mutations are primarily associated with enhanced tumor immunogenicity (high TMB), elevated PD-L1 expression, a proinflammatory TME driven by robust IFN-γ signaling, and increased infiltration of CD4^+^ T cells, CD8^+^ T cells, and B lymphocytes. Notably, the poor efficacy of ICIs is closely associated with a “cold” tumor immune microenvironment characterized by low PD-L1 expression, reduced abundance and functional impairment of CD8^+^ T cells (partly attributed to upregulated TGF-β), decreased infiltration of total PD-1^+^ immune and B cells, increased proportions of tumor-associated macrophages and Tregs, and downregulated expression levels of CXCL10 and CXCL11 (Fig. 1).

### Crosstalk between signaling pathways and immunotherapy

In NSCLC, patients with different oncogenic drivers usually display characteristics of signaling pathways. To improve the effectiveness of immunotherapy, more research is focusing on the crosstalk between signaling pathways and immunotherapy.

### CD73/adenosine pathway

CD73 (Ecto-5′-nucleotidase) is highly expressed in NSCLC with *EGFR* and *KRAS* or *ALK* rearrangements.^104^^,^^105^ Mechanistically, CD73 is regulated by the ERK-Jun pathway, with c-Jun orchestrating CD73 expression by binding to the CD73 genome.^104^ Activation of CD73 catalyzes the dephosphorylation of adenosine monophosphate, converting it into adenosine, thus facilitating evasion of immune surveillance. High CD73 expression indicates a favorable response to ICIs in NSCLC, especially in cases with *EGFR* mutations.^106^ The CD73/adenosine axis is recognized for its role in modulating the immune landscape within the neoplastic milieu, attenuating the immunological engagement of the host with oncogenic cells. Blocking the adenosine pathway may enhance the effectiveness of treatments aimed at stimulating the immune system to attack NSCLC. Given that the CD73/adenosine interplay allows cancer cells to avoid immune detection, further research and clinical trials are required to test the combination of these treatments in patients with this type of lung cancer.^107^^,^^108^

**Figure 2:**
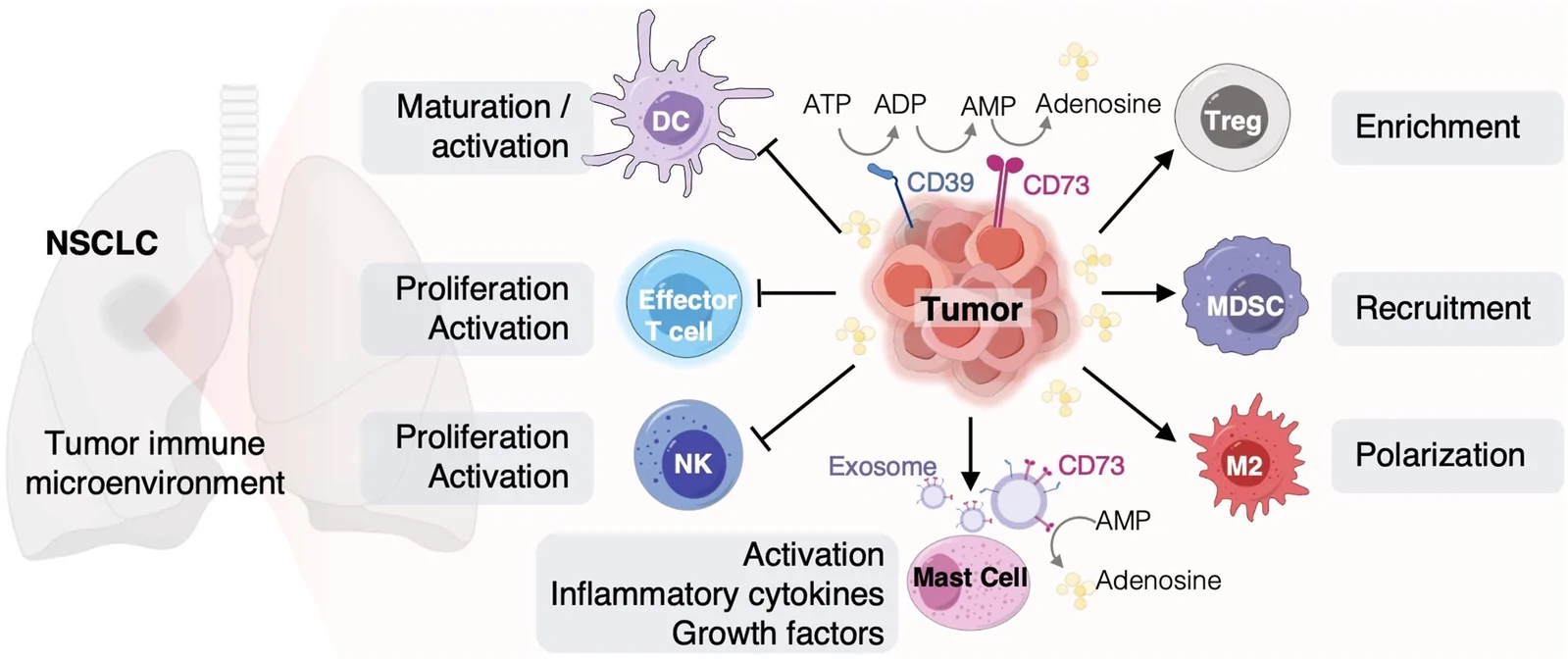
CD73 in NSCLC affects the function of different immune cells in the tumor microenvironment. The novel CD73 antibody Hu001 promotes the proliferation of effector T lymphocytes, increases IFN-γ secretion and NK cell infiltration, and reduces tumor-associated macrophages and MDSCs through adenosine reduction. A novel MMAE-conjugated CD73-ADC promotes the proliferation of T lymphocytes and accumulation of activated dendritic cells, increases IFN-γ secretion, and reduces M2 macrophages in tumors. FOXP3^+^ is significantly increased in NSCLC tumor stroma with high expression of CD73 and CD39. Mast cells could be activated by cancer cells or their released extracellular vesicles, contingent upon the signal transduction of the extracellular enzyme CD73 that mediates the formation of extracellular adenosine and A3 adenosine receptors. Activated mast cells exhibit increased phosphorylation of ERK1/2 MAPK and secretion of IL-8, IL-6, VEGF, and amphiregulin. Note: ADC, antibody–drug conjugate; ERK1/2, extracellular signal-regulated kinase 1/2; FOXP3, fork-head box P3; IFN-γ, interferon-γ; IL, interleukin; MAPK, mitogen-activated protein kinase; MMAE, monomethyl auristatin E; MDSCs, myeloid-derived suppressor cells; NK, natural killer; NSCLC, non-small cell lung cancer; VEGF, vascular endothelial growth factor.

The novel CD73 antibodies Hu001/Hu002 inhibited mouse *Ras*-mutant NSCLC tumor growth via cytotoxicity and TME remodeling. Hu001 enhanced effector T lymphocyte proliferation, increased NK cell infiltration and IFN-γ secretion, and reduced tumor-associated macrophages and MDSCs by decreasing adenosine. Moreover, a novel monomethyl auristatin E-conjugated CD73 antibody–drug conjugate exhibited potent cytotoxicity. It could promote the proliferation of T lymphocytes, the accumulation of activated DCs, increase IFN-γ secretion, and reduce M2 macrophages in tumors.^105^ Activation of CD39 and CD73 exonucleases generates adenosine, which was involved in the establishment of an immunosuppressive TME. This activation was inhibited using separate antibodies to block both membrane-associated and soluble forms of CD39 and CD73, thus blocking the hydrolysis of immunogenic adenosine triphosphate (ATP) into adenosine. These antibodies also promoted anti-tumor immunity by stimulating DCs and macrophages, thereby restoring the activation of T cells. These trends were the foundation for the combination of anti-CD73 and anti-CD39 monoclonal antibodies alongside ICIs.^109^ Mast cells could be activated by cancer cells or their released extracellular vesicles, which was dependent upon the signal transduction of the extracellular enzyme CD73 that mediates the formation of extracellular adenosine and A3 adenosine receptors. Activated mast cells exhibited increased phosphorylation of ERK1/2 MAPK and secretion of IL-8, IL-6, vascular endothelial growth factor, and amphiregulin (Fig. 2).^110^ Notably, in patients with breast cancer and sarcoma, the expression of CD73 was upregulated in tumor-infiltrating NK cells, and the frequency of CD73^+^ NK cells was correlated with tumor size in patients with breast cancer. Compared to CD73^-^ NK cells, immune checkpoint receptors, including LAG-3, V-domain Ig suppressor of T-cell activation (VISTA), PD-1, and PD-L1, were significantly expressed in CD73^+^ NK cells. Moreover, CD73^+^ NK cells underwent transcriptional reprogramming, upregulating IL-10 production through STAT3, thereby inhibiting CD4^+^ T-cell proliferation and IFN-γ production.^111^ Additionally, CD39 and CD73 were associated with tumor development, metastasis, and the inhibition of anti-tumor immune responses.^112^ The combination of anti-PD-L1 and anti-CD73 antibodies significantly inhibited tumor growth by increasing the number of CD8^+^ T cells and enhancing the production of IFN-γ and TNF-α in *EGFR*-mutated NSCLC.^58^

**Figure 3:**
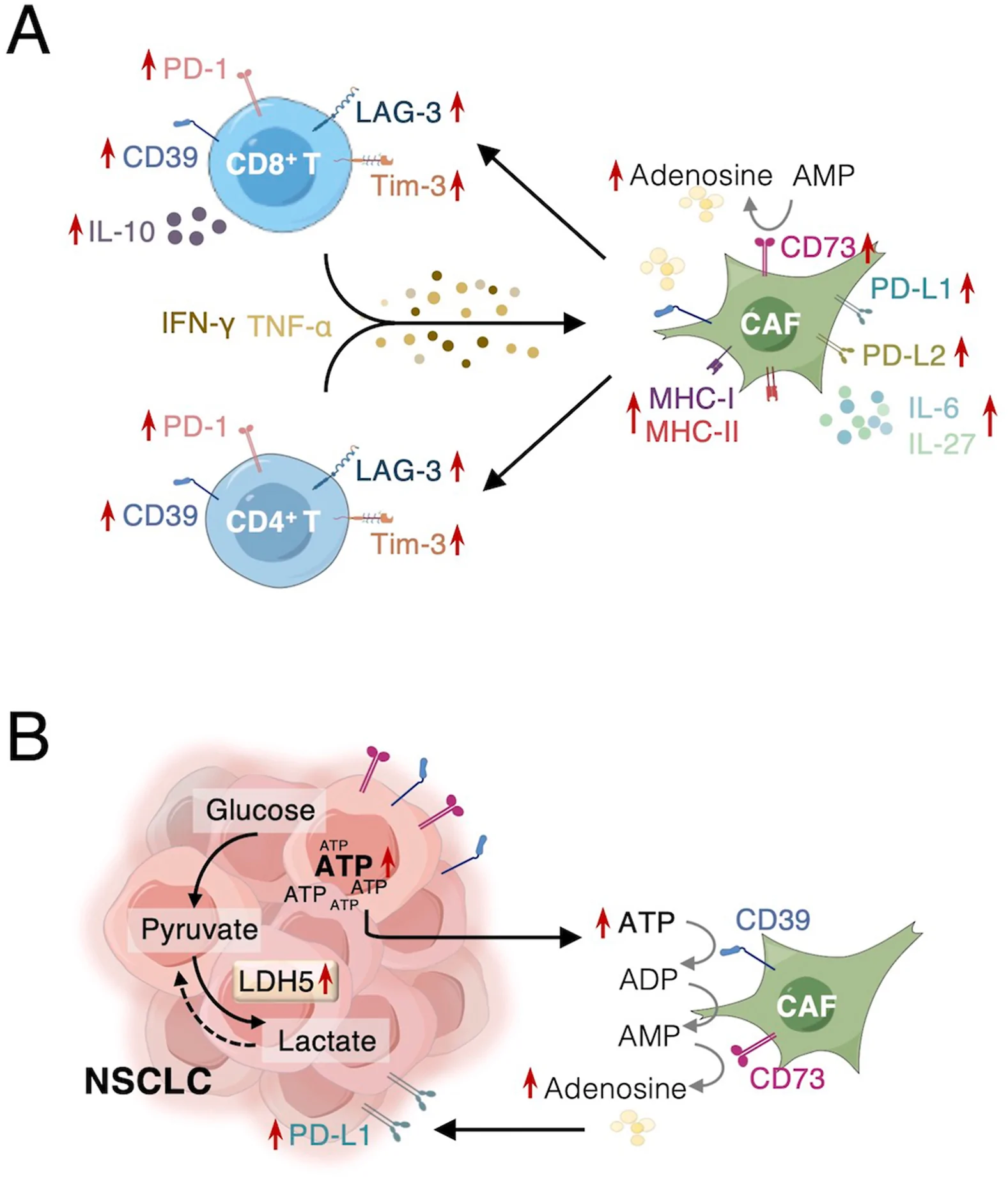
CAFs highly express CD73 and interact with T cells and tumor cells in NSCLC. (A) IFN-γ and TNF-α produced by activated T cells promote the expression of co-inhibitory ligands (PD-L1 and PD-L2) on CAFs, including MHC class I and II levels and CD73, and increase the production of IL-6 and IL-27. In turn, CAFs upregulated co-inhibitory receptors (PD-1, Tim-3, and LAG-3) on T cells alongside the ectonucleotidase CD39 and promote the secretion of IFN-γ by T cells and IL-10 by CD8^+^ T cells in NSCLC. (B) Activated CD73 produces adenosine, inducing the overexpression of LDH5. The large amount of ATP produced by anaerobic glycolysis mediated by LDH5 promotes adenosine production, forming a vicious cycle. The expression of CD39 in CAFs is positively correlated with the expression of PD-L1 in cancer cells. Note: ATP, adenosine triphosphate; CAFs, cancer-associated fibroblasts; IFN-γ, interferon-γ; IL, interleukin; LAG-3, lymphocyte activation gene 3; LDH5, lactate dehydrogenase 5; MHC, major histocompatibility complex; NSCLC, non-small cell lung cancer; PD-1, programmed cell death protein 1; PD-L1, programmed death ligand 1; PD-L2, programmed death ligand 2; Tim-3, T-cell immunoglobulin and mucin-domain containing protein 3; TNF-α, tumor necrosis factor alpha.

IFN-γ and TNF-α produced by activated T cells promoted the expression of PD-L1 and programmed death ligand 2 on CAFs, including MHC molecules and CD73, thereby increasing the production of IL-6 and IL-27. In turn, CAFs upregulated co-inhibitory receptors, including PD-1, Tim-3, and LAG-3, and the ectonucleotidase CD39 on T cells. Additionally, CAFs promoted the secretion of IFN-γ by T cells and IL-10 by CD8^+^ T cells in NSCLC.^113^ CD73 and CD39 were overexpressed in lung cancer cells, CAFs, and TILs. In the matrix of tumors with high CD73 and CD39 expression, the abundance of FOXP3^+^ and PD-1^+^ TILs was significantly increased. Activation of CD73 induced adenosine production, which could subsequently promote the overexpression of lactate dehydrogenase 5 (LDH5). Most of the ATP produced by anaerobic glycolysis mediated by LDH5 promotes adenosine production, resulting in a vicious cycle (Fig. 3).^114^

### NOTCH signaling pathway

The NOTCH signaling pathway is implicated in many aspects of cancer biology, including tumor immune evasion, tumor angiogenesis, metastasis, and cancer stem cell phenotype. Targeting NOTCH signaling may enhance the anti-tumor immune response and sensitize NSCLC tumors to ICI therapy.^115^ Mutations in *NOTCH* may create an inflammatory environment within the tumor, attracting specific immune cells and upregulating genes that increase tumor visibility to the immune system. This mutation profile was linked to an inflammatory gene expression pattern and increased immunogenicity, as evidenced by higher TMB, alterations in DNA damage response genes, and upregulation of MHC-related gene expression.^116^

Patients with NSCLC harboring heightened *NOTCH* signaling mutations experienced a marked improvement in PFS (HR = 0.69; MSKCC cohort) and OS (HR = 0.34; NG cohort) after receiving immunotherapy.^116^ Specific mutations in *NOTCH1-3* and genes involved in DNA repair have been shown to be robust markers of longer PFS (HR = 0.51) with immunotherapy in advanced NSCLC.^117^ Additionally, *NOTCH4* mutations may be predictive of better responses to ICIs due to a high TMB and more active immune markers in pan-cancer.^118^^,^^119^ Similarly, harmful *NOTCH* mutations in patients with NSCLC but without *EGFR/ALK* mutations could be predictive of responsiveness to ICIs due to increased DNA repair and immune-related gene activity. Consequently, combining NOTCH inhibitors with ICI therapy may optimize treatment regimens in clinical practice.^120^ In pancreatic cancer, Notch signaling regulates immunosuppressive macrophages to create an immunosuppressive TME. Combining a NOTCH inhibitor with PD-1 blockade reduced tumor size in mice by enhancing the infiltration of cytotoxic T cells, leading to cancer apoptosis.^121^

### DNA damage signaling pathway

The DNA damage response system comprises eight pathways, namely, mismatch repair (MMR), base excision repair (BER), checkpoint factors, Fanconi anemia, homologous recombination repair (HRR), nucleotide excision repair, nonhomologous end-joining, and DNA translesion synthesis.^122^ These pathways were associated with an immune phenotypic profile characterized by increased TMB, TNB, and immune response-related mRNA expression. Co-mutations involving HRR-MMR or HRR-BER may serve as potential predictive biomarkers for ICB therapy.^123^ Harmful DNA damage response and repair (DDR) mutations are common in NSCLC and are associated with improved clinical prognosis after treatment with PD-L1 blockade.^124^ In NSCLC, co-mutations in the HRR-MMR and HRR-BER pathways were associated with improved survival rates after treatment with atezolizumab.^125^

The absence or inhibition of the DDR pathway may increase immunogenicity due to DNA damage and activate the IFN pathway through cytoplasmic DNA accumulation, ultimately improving the efficacy of immunotherapy.^126^ In an Lewis lung carcinoma mouse model, knocking out poly ADP-ribose polymerase 1 (PARP1) may activate T cells and induce T lymphocyte-mediated tumor growth control.^127^

After treatment with poly ADP-ribose polymerase (PARP) inhibitors, homologous recombination-deficient tumors suffered significant levels of DNA damage following the increase in TMB, which promoted the release of new antigens and upregulated the expression of PD-L1. A prospective phase II study provided evidence of the safety and antitumor activity of the combination of niraparib (a PARP1 and PARP2 inhibitor) and dostarlimab (PD-1 inhibitor) in patients with HRR gene-mutant advanced NSCLC with positive PD-L1 expression.^128^ PARP1 could detect and bind to sites of DNA damage, and niraparib combined with pembrolizumab demonstrated clinical activity in patients with advanced and/or metastatic NSCLC.^129^ Mutant *LKB1* led to defects in DNA damage repair processes and abnormal activation of PARP1, which impaired the IFN-γ pathway. PARP1 inhibition restored the disrupted IFN-γ signaling and converted “cold” tumors into “hot” tumors, providing a theoretical basis for the dual inhibition therapy of PARP and PD-1 in *LKB1* mutant NSCLC.^130^ Following chemotherapy and ROS1 inhibitor treatment, a LUAD patient with *ROS1* fusion and HRR gene mutations was shown to exhibit disease progression. However, the combination of sintilimab (PD-1 inhibitor) and niraparib improved the PFS and OS of this cohort.^131^

### STING signaling pathway

Activation of the cGAS-STING pathway triggers the production of type I interferons, activates DCs, and primes CD8^+^ T cells to recognize and respond to tumor-associated antigens.^132^^,^^133^

Agonists of the stimulator of interferon genes induced type I interferon production and increased PD-L1 expression, suggesting that agonists combined with ICIs may produce a beneficial outcome.^134^ ALG-031048, a second-generation STING agonist, improved anti-tumor efficacy when used with ICIs in a mouse model of colon cancer.^135^ CGAS catalyzes the synthesis of cGAMP, which activates the STING protein to induce the production of type I IFN. When used in combination with a PD-L1 antibody, cGAMP effectively suppressed tumor growth in mice with B16 melanoma tumors.^136^ In a B16–F10 lung metastasis model, a STING agonist enhanced the anti-tumor effects of anti-PD-1 by increasing the mRNA levels of CD3, CD4, NK1.1, PD-1, and IFN-γ, as well as activating NK cells.^137^

The combination of MIW815 (STING agonist) and spartalizumab (PD-1 inhibitor) exhibited good tolerability in patients with advanced/metastatic cancer. However, the effectiveness of this treatment was limited.^138^ Repeated intra-tumoral injection of SYNB1891 to activate the STING pathway, in combination with atezolizumab, was safe and promising for advanced malignancies.^139^ MSA-2 (STING agonist) reshaped the immune microenvironment of cervical cancer. The combination of MSA-2 and anti-PD-1 antibody was expected to improve prognosis compared to anti-PD-1 monotherapy.^140^ Although the combination of STING agonists and ICIs exhibited effectiveness in tumors, further investigations are necessary to address the limited efficacy due to pathway silencing and the potential for immune cytokine storms resulting from pathway overactivation.^141^

### Wingless-related integration site (Wnt)/β-catenin signaling pathway

The Wnt/β-catenin pathway is essential for cell growth and survival and is often disrupted in NSCLC. This outcome promotes immune evasion and resistance to ICIs. Activation of the Wnt/β-catenin pathway in NSCLC may lead to the upregulation of immune checkpoint molecules, suppression of anti-tumor immune responses, and enhancement of tumor growth and metastasis. Thus, targeting the Wnt/β-catenin signaling pathway in combination with ICIs may represent a promising approach to overcoming immune evasion mechanisms and enhancing the effectiveness of immunotherapy in NSCLC.^142^

Compared to T-cell-inflammatory tumors, non-T-cell-inflammatory tumors exhibited three times more mutations in the β-catenin signaling molecule. Therefore, it is necessary to develop inhibitors of this pathway to restore immune cell infiltration and enhance immunotherapy.^143^ Activation of Wnt/β-catenin signaling impaired CD8^+^ T-cell infiltration. The combination of PD-1 blockade and Wnt/β-catenin signaling inhibitors induced better anti-tumor immunity than either treatment alone.^144^ Overactive β-catenin signaling also inhibited the recruitment of DCs, promoted CD8^+^ T-cell exclusion, increased the number of Treg cells, and induced the expression of PD-L1 and PD-1. Therefore, the combined inhibition of Wnt/β-catenin and anti-PD-L1 may induce durable anti-tumor immunity.^145^

Proximal Wnt ligand signaling activity was associated with resistance to anti-PD-1. In a mouse tumor model, Wnt signaling was shown to induce kynurenine production and polymorphonuclear myeloid-derived suppressor cell accumulation, whereas Wnt ligand inhibition enhanced the anti-tumor effect of PD-1 blockade.^146^ Blocking Wnt/β-catenin signaling could directly inhibit tumor proliferation and migration, promote T-cell infiltration and PD-L1 expression, and enhance the efficacy of PD-1 blockade in glioblastoma.^147^ Downregulation of Wnt/β-catenin may enhance the sensitivity of gastric cancer (GC) cells to PD-1 blockade *in vitro*. Double targeted inhibition of β-catenin and PD-1 may be a potentially effective treatment for patients with GC.^148^ Wnt signaling was activated in pancreatic ductal adenocarcinoma, while CD4^+^ T-cell infiltration in the TME induced T-cell factor 7 (TCF7) in human pancreatic cancer. After conditionally inactivating TCF7 on CD4^+^ T cells in a mouse pancreatic cancer model, CD8^+^ T cells increased, and Treg cells decreased. In contrast, the expression of PD-L1 was upregulated in the TME. In the experimental model, pharmacological Wnt signaling inhibition improved the efficacy of PD-L1 blockade.^149^

### Tumor necrosis factor α signaling pathway

Mutations in TNF-α signaling have been identified as a promising clinical biomarker for ICI therapy in NSCLC, correlating with a markedly prolonged OS. This association was attributed to elevated immunogenicity, reflected by increased TMB, TNB, and mutations in DNA damage response signaling, alongside a higher density of tumor-infiltrating immune cells.^150^ TNF-α could bind to tumor necrosis factor receptor 1 (TNFR1) and TNFR2. The transmembrane form of TNF-α (tmTNF-α) preferentially binds to TNFR2. TmTNF-α is cleaved by TNF-alpha-converting enzyme (TACE), resulting in the release of its soluble form TNF-α, which has a higher affinity for TNFR1. The expression levels of TNF and TACE were significantly reduced in patients with LUAD, while the balance of TNFR1/TNFR2 was elevated. Therefore, TNF-α may not serve as a potential biomarker for immunotherapy, but TACE may play a crucial role.^151^ The combination of anti-TNFR2 and anti-PD-L1 antibodies could eradicate tumors, prolong the OS of patients with pancreatic cancer, activate CD8^+^ T cells, and reduce the infiltration of Treg cells and tumor-related macrophages. Overall, this cascade of events culminated in strong anti-tumor immune memory. The anti-tumor immune response mainly relied on CD8^+^ T cells, partially on CD4^+^ T cells, and not on NK cells (Table 2).^152^

**Table 2:** Signaling pathways responsible for remodeling the immune microenvironment in cancer.

Signaling Pathway	Tumor Type	TME Remodeling Features	TME Alteration After Treatment	Mechanism	References
CD73/Adenosine	NSCLC	Upregulation of FOXP3^+^, PD-1^+^ TILs Activation of mast cells Increased of IL-6, IL-8, IL-10, IL-27, VEGF, and amphiregulin Upregulation of MHC molecules, PD-L1, PD-L2 on cancer associated fibroblasts	CD73-targeted antibody: Upregulation of DCs, proinflammatory macrophages, NK cells, and effector T cells Downregulation of M2 macrophages, and MDSCs Secretion of IFN-γ CD73-targeted antibody combined with anti PD-L1 antibody: Upregulation of CD8^+^ T cells Increased IFN-γ and TNF-α	ERK-Jun CD73 Adenosine monophosphate adenosine Cancer cells or extracellular vesicles activated mast cells IL-8, IL-6, VEGF, and amphiregulin	58,104, 105, 106,110,113,114
	Breast cancer and sarcoma	Upregulation of LAG-3, VISTA, PD-1 and PD-L1 on CD73^+^ NK cells Downregulation of CD4^+^ T cell Increased IL-10, decreased IFN-γ	NA	CD73^+^ NK STAT3 increased IL-10, decreased IFN-γ	111
NOTCH	NSCLC	Upregulation of activated memory CD4^+^ and CD8^+^ T cells, M0 and M1 macrophages, antigen-processing machinery Upregulation of TMB, MHC-related genes, PD-1, PD-L1, and CTLA4	NA	Increased immune-related gene activity Transcription of genes associated with DDR	116,118,120
	Melanoma	Upregulation of activated CD4^+^, CD8^+^ T cells, Tfh cells, and neutrophils Upregulation of TMB and TNB	NA	Higher expression levels of immune-related genes Increased interferon IFN-γ response and DDR mutations	119
	Pancreatic cancer	Decreased macrophage infiltration Higher levels of immunosuppressive mediator in TAMs	NOTCH inhibition combined with anti PD-1 antibody: Increased cytotoxic T-cell infiltration	NA	121
DNA damage	NSCLC	Upregulation of activated T cells	PARP inhibition: Upregulation of PD-L1, TMB Restored the disrupted IFN-γ signaling	NA	127,128,130
	Pan cancer	Upregulation of PD-L1, TMB, and TNB	NA	Increased mRNA expression related to immune response Activation of the IFN pathway	123,126
cGAS–STING	Pan cancer	Upregulation of activated CD8^+^ T cells, DCs Increased PD-L1 expression	NA	Triggering the production of type I interferon	132, 133, 134
	Melanoma	NA	Intramuscular delivery of cGAMP: Activated DCs Stimulated cross-presentation of tumor-associated antigens STING agonist: Increased mRNA levels of CD3, CD4, NK1.1, PD-1 and IFN-γ Activated NK cells	Triggering the production of type I interferon	136,137
	Cervical cancer	NA	STING agonist: Upregulation of T cells, NK cells Increased intercellular communication within immune response pathways	Triggering the production of type I interferon	140
Wnt/β-catenin	NSCLC	Downregulation of CD8^+^ T cell	NA	NA	144
	Pan cancer	Upregulation of Treg cells Inhibited the recruitment of DCs and CD8^+^ T cell infiltration Upregulation of PD-L1 and PD-1	Wnt ligand inhibition: Decreased local Kyn levels Suppressed PMN-MDSC recruitment Wnt ligand inhibition combined with anti PD-1 antibody: Upregulation of CD8^+^ T cell	NA	145,146
	Pancreatic ductal adenocarcinoma	Upregulation of CD8^+^ T cell, PD-L1	NA	NA	149
		Downregulation of Treg cells			
Tumor necrosis factor α	NSCLC	Upregulation of activated memory CD4^+^, CD8^+^ T cells and Tfh cells Downregulation of Treg cells Upregulation of TMB, TNB, PD-L1, LAG3 and CD276	NA	Transcription of genes associated with DDR	150
	Pancreatic ductal adenocarcinoma	Upregulation of PD-L1 Suppressed cancer immunogenicity	TNFR2 blockade combined with anti PD-L1 antibody: Upregulation of activated CD8^+^ T cells Downregulation of Treg cells and TAMs	TNFR2 p65 NF-κB PD-L1	152

Note: CTLA4, cytotoxic T lymphocyte antigen 4; DCs, dendritic cells; DDR, DNA damage response; ERK, extracellular signal-regulated kinase; FOXP3, fork-head box P3; IFN-γ, interferon-γ; IL, interleukin; Kyn, kynurenine; MDSCs, myeloid-derived suppressor cells; MHC, major histocompatibility complex; NK, natural killer; NSCLC, non-small cell lung cancer; PARP, poly ADP-ribose polymerase; PD-1, programmed cell death protein 1; PD-L1, programmed death ligand 1; PD-L2, programmed death ligand 2; PMN-MDSC: polymorphonuclear myeloid-derived suppressor cell; TAMs, tumor-associated macrophages; Tfh cells, follicular helper T cells; TILs, tumor infiltrating lymphocytes; TMB, tumor mutation burden; TME, tumor microenvironment; TNB, tumor neoantigen burden; TNF-α, tumor necrosis factor alpha; TNFR2, tumor necrosis factor receptor 2; Treg cells, regulatory T cells; VEGF, vascular endothelial growth factor; VISTA, V-domain Ig suppressor of T cell activation.

### PI3K/AKT signaling pathway

The PI3K/AKT signaling axis plays a crucial role in oncogenic processes, including cellular proliferation, apoptosis resistance, and metabolic regulation. Enhanced PI3K/AKT pathway activity has been implicated in the pathogenesis of NSCLC harboring *EGFR* exon 20 insertions; this pathway contributed to the emergence of immune escape mechanisms observed within this neoplasm. Therefore, targeting the PI3K/AKT pathway in combination with other therapeutic strategies, such as ICIs, offers a potential approach to overcoming immune tolerance and enhancing anti-tumor immunity in NSCLC with *EGFR* exon 20 insertions.^153^ The PI3K/AKT pathway mutation status was an independent predictor for the response of patients with colon adenocarcinoma (COAD) to ICI treatment. Patients with COAD with PI3K/AKT mutations had a better OS, higher immunogenicity, higher immune response scores, and increased immune cell activation.^154^

### Biological markers for immunotherapy

A discovery cohort identified elevated CD34 expression as a novel biomarker for anti-PD-1 therapy, showing reduced PFS (HR = 5.011) with camrelizumab (PD-1 inhibitor).^155^

Increasing attention has been given to the role of the tumor microbiome in cancer immunotherapy, particularly the gut microbiota in melanoma and NSCLC.^156^, ^157^, ^158^ Intra-tumoral Escherichia was associated with longer OS (16 *vs.* 11 months; HR = 0.73) in advanced NSCLC patients treated with single-agent ICI but not CIT.^159^

### Countermeasures for immunotherapy resistance

Immunotherapy (PD-1/PD-L1 inhibitors), alone or combined with chemotherapy, is the standard treatment for newly diagnosed advanced or metastatic NSCLC. However, immunotherapy resistance has spurred investigations into subsequent treatment options. Chimeric antigen receptor (CAR) T-cell therapy holds great promise for NSCLC, with EGFR, mesothelin, prostate stem cell antigen, mucin 1, and CD98 heavy chain protein emerging as promising targets.^160^^,^^161^ In addition to CAR T-cell therapies, immune co-stimulatory antibodies, T-cell agonists, oncolytic viruses, vaccines, and TIL therapies are under development to identify more effective therapies after immunotherapy resistance.^162^

## Conclusions

In this review, we have synthesized existing evidence to propose that the TME in NSCLC can be stratified into distinct immunological phenotypes based on the underlying oncogenic driver mutations. We recommend a comprehensive baseline assessment including next-generation sequencing for core driver mutations (*e.g.*, *KRAS*, *EGFR*, *BRAF*, *ALK*, *HER2*) and PD-L1 expression to guide initial therapy selection. *KRAS* mutations (including G12C, G12C/TP53 co-mutations, and rare variants): ICIs are recommended. Uncommon EGFR mutations (*e.g.*, G719X, L861Q, S768I, exon 20 insertions): PD-1 blockade may be considered. *BRAF*^*non-V600E*^ mutations: First-line ICIs are recommended if targeted therapy is unavailable or after resistance develops. *ALK* rearrangements: First-line ICI monotherapy is not recommended; combination with chemotherapy is preferred to enhance efficacy. *HER2* mutations: Either ICI monotherapy or CIT may be beneficial, although outcomes are often poor in those with exon 20 insertion mutations.

For patients who are unresponsive to initial therapy, repeat testing of next-generation sequencing and biomarker status (*e.g.*, PD-L1 expression, CD34 and CD73 levels, and signaling pathway-related markers) is recommended to guide treatment regimen modification. The combination of adagrasib and pembrolizumab is a promising therapeutic strategy for patients with the *KRAS* G12C isoform. Following EGFR-TKI failure, patients with G719X mutations, exon 20 insertions, positive p53 expression, or low AXL expression should be indicated for ICI monotherapy. However, the combination of EGFR-TKIs and immunotherapy is associated with adverse effects, hindering its clinical application. For patients without T790M mutations, ICI plus chemotherapy is recommended. For patients with *BRAF*^*V600E*^ mutations who progress after TKI treatment, ICI-based combination therapies are suggested. Combinations involving ALK-TKI and ICI are effective regardless of prior ALK-TKI treatment, although treatment-naive patients tend to exhibit better ORR. Moreover, enhanced CD34 expression and gut microbial features, particularly increased intratumoral *Escherichia* abundance, are recommended as auxiliary predictive markers for ICI responsiveness. Activation of the STING pathway, as well as inhibition of NOTCH, CD73/adenosine, TNF-α, Wnt/β-catenin, the DNA damage response, and PI3K/AKT signaling, can contribute to remodeling the TME and transforming immunologically “cold” tumors into therapy-responsive phenotypes, offering novel therapeutic avenues to enhance the efficacy of combination immunotherapy.

In conclusion, the integration of genetic subtyping with TME profiling is paramount for advancing NSCLC immunotherapy. The implementation of the strategies outlined here, coupled with ongoing basic and clinical research, holds the promise of achieving more durable responses for a broader patient population.

## CRediT authorship contribution statement

**Chunyan Liu:** Writing – review & editing, Writing – original draft, Visualization, Conceptualization. **Shan Zhu:** Writing – review & editing, Visualization, Funding acquisition. **Jing Wu:** Writing – review & editing, Funding acquisition. **Yuan Qiao:** Writing – review & editing. **Ning Yang:** Writing – review & editing, Funding acquisition. **Jingtao Chen:** Writing – review & editing, Writing – original draft, Supervision, Funding acquisition, Conceptualization.

## Funding

This work was supported by the 10.13039/501100012166National Key Research and Development Program (China) (No. 2021YFF0704800), the 10.13039/501100001809National Natural Science Foundation of China (No. 82273186, 82102714), the Scientific and Technological Developing Plan of 10.13039/501100003807Jilin Province (China) (No. YDZJ202201ZYTS098), the Jilin Provincial Department of Education Scientific Research Project (China) (No. JJKH20231213KJ), and the Jilin Provincial Specialized Fund for Medical and 10.13039/100018696Health Talents (China) (No. JLSWSRCZX2023-111).

## Conflict of interests

The authors declare that they have no conflict of interests.
